# Supplementary material for: Synthesis of Analogs to A-Type Proanthocyanidin Natural Products with Enhanced Antimicrobial Properties against Foodborne Microorganisms
Source: Molecules. 2023 Jun 19;28(12):4844. doi: 10.3390/molecules28124844 (PMC10302345; doi:10.3390/molecules28124844)
Supplement: Supplementary file 1 [file molecules-28-04844-s001.zip › molecules-2445146-supplementary.pdf]

Table S1. Checkerboard assay of compound analogue **4** together with remaining compounds against *Staphylococcus saprophyticus* UJA27g.

	MIC of each agent (μg/mL)				
**Analogue**	**Alone**	**Combination**	**FIC**	**FICI**	**Outcome**
**4**	10	1.25	0.125		
**1**	10	5	0.5	0.625	IND
**4**	10	2.5	0.25		
**2**	1000	250	0.25	0.5	SYN
**4**	10	1.25	0.125		
**3**	10	5	0.5	0.625	IND
**4**	10	1.25	0.125		
**5**	10	2.5	0.25	0.375	SYN
**4**	10	1.25	0.125		
**6**	10	5	0.5	0.625	IND
**4**	10	1.25	0.125		
**7**	10	10	1	1.125	IND

MIC: minimal inhibitory concentration; FIC: fractional inhibitory concentration (FIC=MIC combination/MIC alone); FICI= FIC of analogue **4** + FIC of other compound.

SYN: synergy. IND: indifferent.

Table S2. Checkerboard assay of analogue **4** together with remaining compounds against *Bacillus cereus* UJA27q.

	MIC of each agent (μg/mL)				
**Analogue**	**Alone**	**Combination**	**FIC**	**FICI**	**Outcome**
**4**	50	6.25	0.125		
**1**	50	25	0.5	0.625	IND
**4**	50	6.25	0.125		
**2**	50	25	0.5	0.625	IND
**4**	50	6.25	0.125		
**3**	50	50	1	1.125	IND
**4**	50	6.25	0.125		
**5**	1000	500	0.5	0.625	IND
**4**	50	6.25	0.125		
**6**	10	5	0.5	0.625	IND
**4**	50	6.25	0.125		
**7**	10	5	0.5	0.625	IND

MIC: minimal inhibitory concentration; FIC: fractional inhibitory concentration (FIC=MIC combination/MIC alone); FICI= FIC of analogue **4** + FIC of other compound.

IND: indifferent.

Table S3. Checkerboard assay of analogue **4** together with remaining compounds against *Staphylococcus aureus* UJA34f.

	**MIC of each agent (µg/mL)**				
**Analogue**	**Alone**	**Combination**	**FIC**	**FICI**	**Outcome**
**4**	50	6.25	0.125		
**1**	10	5	0.5	0.625	IND
**4**	50	6.25	0.125		
**2**	10	5	0.5	0.625	IND
**4**	50	12.5	0.25		
**3**	50	12.5	0.25	0.5	SYN
**4**	50	6.25	0.125		
**5**	10	5	0.5	0.625	IND
**4**	50	6.25	0.125		
**6**	10	2.5	0.25	0.375	SYN
**4**	50	6.25	0.125		
**7**	10	2.5	0.25	0.375	SYN

MIC: minimal inhibitory concentration; FIC: fractional inhibitory concentration (FIC=MIC combination/MIC alone); FICI= FIC of analogue **4** + FIC of other compound.

SYN: synergy. IND: indifferent.
